# Modern Dental Materia Medica, Pharmacology and Therapeutics

**Published:** 1910-03-15

**Authors:** 


MODERN DENTAL MATERIA MEDICA, PHARMACOLOGY AND THERAPEUTICS. This is the comprehensive title of another contribution to dental medicine by J. P. Buckley, Ph.G., D.D.S., Professor of Materia Medica and Therapeutics in the Chicago College of Dental Surgery. It is a nicely made book of nearly 400 pages, published by P. Blakiston's Son & Co., Philadelphia. The author in his preface states that he has endeavored to incorporate in his work "all that a dentist should know about drugs and remedies and their practical application in the treatment of disease." We wish he had not put this statement in his preface, as it makes one feel as though the last word on the subject has been said, which in our judgment would be calamitous. The work contains a very comprehensive statement of both drugs and physical remedies. In the first part the line of treatment is to give the materia medica, pharmacology and the therapeutics of each drug much the same as style as it is given in the Dispensatory. The classification is much simplified and very practical under the following general heads: Local Remedies, General Remedies, and Remedies other than Drugs. The discussion of the remedies is on the classified order and little personality is thus permitted to influence the statements. This part of the work is preceded by a brief statement of the general sources and preparations of remedies from crude drugs, methods of administration, and physical conditions modifying drug reactions. At the close of the first part the principles and art of prescribing are well set forth.

The second part is devoted to Practical Dental Therapeutics and here of course will center the interest of the dental practitioner, and here the author has a chance to put his own ideas into the practical treatment.

This part the author has confined almost entirely to local affections and local treatment. He makes but two general heads for his classification: First, diseases of the teeth and the soft tissues immediately associated; and diseases of the soft tissues of the mouth not intimately connected with the teeth.

The author states that the first essential factor in the treatment of disease successfully, is to be able to make a proper diagnosis of the condition. Just here we think he should have laid down the principles at least involved in making a diagnosis in the mouth, instead of repeating the specific technique in connection with his treatment of diseases later on in the work. Very few practitioners know how to systematically diagnose dental disturbances, because our books and instructors don't give us the principles to start with. Of course all this would involve a good knowledge of tissues in health and also their peculiar diseases to make it of sufficient value to direct treatment. This would probably increase the bulk of matter in the book to an undesirable extent. We can not, however, make intelligent use of remedies without this knowledge and it would seem of such importance as to warrant its incorporation in any book on therapeutics. The treatment of infected pulp canals, alveolar abscess and associated diseases is a restatement of the authors methods so well known and proven as to be very generally accepted. We had hoped the author would present the pathology of treatment of peridental diseases in a complete and classified manner. About twenty pages are given to the treatment of pyorrhoea alveolaris, which does not cover the subject satisfactorily. This is one of the livest questions being discussed by the profession everywhere to-day and we are all seeking new and better light on this most difficult problem. Much data is already available but it will probably be some time before it can be classified for a text book. We do not find in any of these new works on dental medicine a complete statement of oral prophylaxis treatment. It seems to be a generally prevailing idea that this treatment is so simple that no one feels the need of a serious statement of its technical principles. Dr. Buckley is practicing this art and knows of its great value and we do not believe his book would have suffered had it contained a dignified statement of the details of the surgical and technical methods used. We have so many inquiries as where these details can be found that it would, we are sure, be a much appreciated addition to any work on this subject. It belongs in a treatise on Dental therapeutics rather than in a work on operative Dentistry.

Dr. Buckley has made a book that will be handy for quick reference because of its practical classification and the terseness of his statements will appeal to many, and we have no doubt but it will meet with a large sale. It is well made from the printers standpoint.

